# Reproducible and Sustained Regulation of Gαs Signalling Using a Metazoan Opsin as an Optogenetic Tool

**DOI:** 10.1371/journal.pone.0030774

**Published:** 2012-01-24

**Authors:** Helena J. Bailes, Ling-Yu Zhuang, Robert J. Lucas

**Affiliations:** Faculty of Life Sciences, The University of Manchester, Manchester, United Kingdom; Pennsylvania State University, United States of America

## Abstract

Originally developed to regulate neuronal excitability, optogenetics is increasingly also used to control other cellular processes with unprecedented spatiotemporal resolution. Optogenetic modulation of all major G-protein signalling pathways (Gq, Gi and Gs) has been achieved using variants of mammalian rod opsin. We show here that the light response driven by such rod opsin-based tools dissipates under repeated exposure, consistent with the known bleaching characteristics of this photopigment. We continue to show that replacing rod opsin with a bleach resistant opsin from *Carybdea rastonii*, the box jellyfish, (JellyOp) overcomes this limitation. Visible light induced high amplitude, reversible, and reproducible increases in cAMP in mammalian cells expressing JellyOp. While single flashes produced a brief cAMP spike, repeated stimulation could sustain elevated levels for 10s of minutes. JellyOp was more photosensitive than currently available optogenetic tools, responding to white light at irradiances ≥1 µW/cm^2^. We conclude that JellyOp is a promising new tool for mimicking the activity of Gs-coupled G protein coupled receptors with fine spatiotemporal resolution.

## Introduction

G protein coupled receptors (GPCRs) are the largest family of metazoan cell surface receptors and contribute to inter- and intra-cellular communication in all major body systems. They are also important therapeutic targets, with as many as a third of top-selling pharmaceutical drugs regulating their activity[1]. Improved tools to regulate GPCR activity are thus of great interest from both experimental and therapeutic perspectives.

Optogenetics is one of the most exciting recent technological developments in neuroscience. In brief, cell types of interest are engineered to express light-sensitive proteins (photopigments), allowing light to be used to regulate their activity remotely and with unprecedented spatial and temporal precision. The most widely used photopigments in optogenetics are microbial light-gated ion channels, which have been extensively exploited to allow direct control of neuronal membrane potential (e.g. [2], [3], [4] for reviews). More recently, attention has turned to non-excitable cells, and to photopigments capable of regulating cellular systems other than ionic permeability.

The opsin photopigments that support vision across the animal kingdom are GPCRs whose signalling activity is regulated by light. They thus represent natural candidates for optogenetic control of G-protein signalling. *Drosophila* Rh1 and, more recently, mammalian melanopsin have been used to provide photic control of Gαq signalling [5], [6]. However, the most flexible tools in this area have been based upon rod opsin, the photopigment of vertebrate rods. Rod opsin's cognate G-protein is transducin, a member of the Gαi subclass, and it has been exploited to regulate Gαi signalling for experimental purposes [7], [8]. However, thanks to the extensive structural information available for this protein, chimeric receptors (recently termed OptoXRs) comprising the light sensitive elements of rod opsin fused to intracellular signalling components of other GPCRs have been used to gain access also to the third major class of G protein, Gαs [9], [10].

Rod opsin has many advantages as a starting point for designing optogenetic tools: 1.) It expresses efficiently in non-native environments without adding specialist chaperones/folding factors. 2.) Its signalling characteristics are well described and specific [11], [12]. 3.) Its basic structure is well-defined, as are many structure:function relationships [13], [14], [15], [16], facilitating the design of chimera and site-directed mutants aimed at optimising its use for particular cell types and modifying its signalling activity. However, it has one potentially important limitation - its reliance upon *cis*-isoforms of retinaldehyde to recover from bleach. In the vertebrate retina an enzymatic pathway ensures a steady supply of such *cis*-isoforms, but this pathway is absent elsewhere in the mammalian body. It seems likely then that the availability of *cis*-retinaldehyde would limit the magnitude of rod-opsin driven responses outside of the eye. Moreover, while application of exogenous *cis*-isoforms could alleviate this problem, the effect of such an approach should steadily decrease during light exposure as *cis*-isoforms are degraded. Previous analyses of OptoXRs have not probed this behaviour because they have relied upon either end-point assays of second messenger or single stimulation protocols [7], [10]. Here, using a real time reporter for cAMP, we present evidence that pigment bleach does indeed impose a fundamental limit on the magnitude and reproducibility of rod opsin-based OptoXR activity.

Many opsins from invertebrates (and a very few from vertebrates) do not bleach like rod opsin. These so-called bistable pigments bind both *cis*- and *trans*- isoforms of retinaldehyde, with light driving isomerisation in both directions. This comprises an intrinsic bleach recovery mechanism that makes bistable pigments much less reliant upon a supply of *cis*-retinaldehydes. Such bistable pigments could therefore provide improved tools for optogenetic control, allowing higher amplitude and more reproducible light responses. Here we show that a bleach-resistant opsin from the box jellyfish, *Carybdea rastonii,* previously shown to be naturally Gαs-coupled [17], does indeed allow much superior control of G protein signalling. The light dependent increase in cAMP induced by this ‘JellyOp’ is both higher amplitude and, especially, more repeatable than the response driven by currently available OptoXRs. As we also show that JellyOp responds to modest levels of visible light it represents an accessible tool for optogenetic control of GPCR signalling.

## Methods

### Construction of receptors

**Figure 1:**
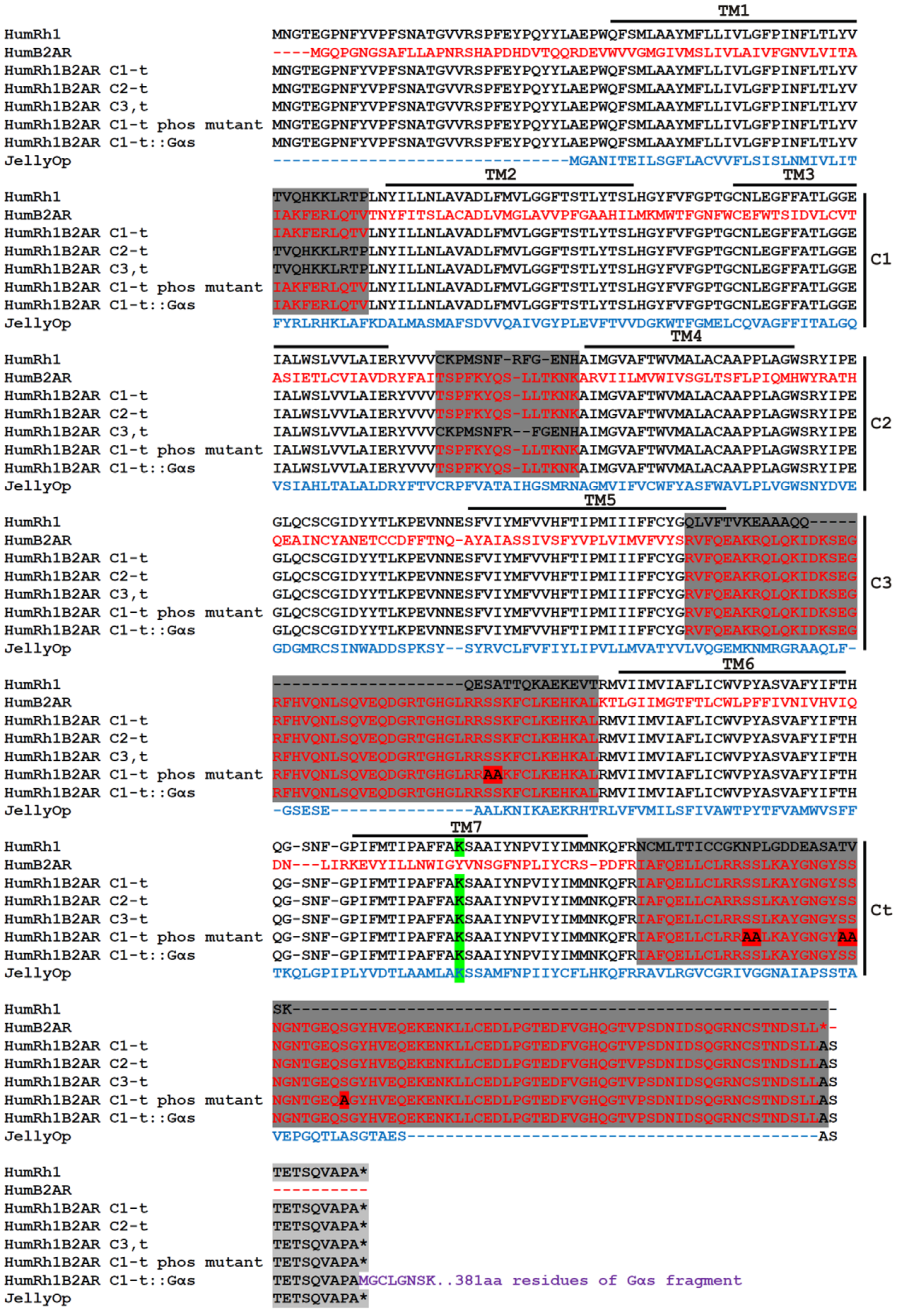
OptoXR sequence alignment. A number of structural variants on a published OptoXR chimera comprising elements of the human β2AR and human rhodopsin sequences were generated in an attempt to increase response amplitude/reproducibility. An amino acid alignment of these variants and, for comparison the human β2AR and rhodopsin (Genbank NM_000539.3, in red and NM_000024, in black), and JellyOp (Genbank AB435549, in blue) sequences are shown. Structural boundaries are based on bovine rhodopsin [14] with putative cytoplasmic regions shaded in dark grey. The lysine residue in TM7, which forms a Schiff-base linkage with retinaldehyde chromophore, is highlighted in green. Note that the terminal 9 amino acids of rod opsin are included as an epitope tag (1D4) in all receptors used in this study (light grey shading). In addition to the published OptoXR in which the entire cytoplasmic surface of rod opsin is replaced by that of the β2AR (Rh1B2AR 1-t), variants in which either 1^st^ or 1^st^ and 2^nd^ intracellular loops from rod opsin were retained (Rh1B2AR 2-t and Rh1B2AR C3,t) in the hope of improving chimera stability were generated. Other variants on Rh1B2AR 1-t employed site directed mutagenesis of phosphorylation sites (highlighted in red) important for arrestin binding and receptor inactivation [18] (Rh1B2AR 1-t phos mutant) or a fusion of the human Gαs subunit at the C-terminal tail in purple (Rh1B2AR 1-t::Gαs).

Construction of chimeric receptors was carried out following the optimal cytoplasmic boundaries described by Kim et al (2005)[9]. Our version of the ‘standard’ receptor [9], [10] was designed from human rhodopsin and β2AR sequences (Genbank NM_000539.3 and NM_000024, respectively; Figure 1) to maximise any future therapeutic potential of the protein. The sequence also included silent substitutions to introduce unique restriction enzyme recognition sites within each transmembrane region to facilitate cloning different cytoplasmic loops. The nucleotide sequence for Rh1β2AR 1-t with silent substitutions was synthesised by Genscript Corp and supplied in puc57 cloning vector. A phosphorylation mutant (Rh1β2AR 1-t phos mutant) was engineered by exchanging serine residues (7 in total; 261, 262, 345, 346, 355, 356, 364 based on the numbering of residues of the human β2AR) known to be important for PKA and GRK phosphorylation for alanine (following [18]), using site directed mutagenesis (lightning quikchange kit; Stratagene). In addition, a mutant was created from the standard Rh1β2AR 1-t (termed Rh1β2AR 1-t::Gαs) with the stop codon replaced with a 6-base linker and the coding region for a ‘short’ transcript of the human Gαs protein (Figure 1). The transcript on which the coding region for Gαs was based (GNASS; Genbank NM_080426.2) has been shown to have less constitutive activity than longer forms [19]. A 1D4 epitope was fused at the 3′ end of every receptor construct coding region, composed of the last 9 amino acid codons from bovine rhodopsin followed by a stop codon. All sequences were verified via standard sequencing techniques prior to use. The coding regions of the receptors used were flanked by HindIII and NotI sequences and cloned into the mammalian expression vector pcDNA3.1. The sequence for the opsin of the box jellyfish *Carybdea rastonii* (Genbank AB435549; JellyOp) was also synthesised by Genscript Corp in a puc57 vector and fused with a 1D4 epitope. An HpaI recognition site was introduced into the multiple cloning site of the pcDNA3.1 vector and the jellyfish sequence flanked with HpaI and NotI sites for cloning into pcDNA3.1-HpaI. A jellyfish lysine mutant plasmid was also engineered using site directed mutagenesis to alter the putative chromophore binding site at residue 296 from lysine to alanine (numbering from bovine rhodopsin [14]).

### cAMP reporter

**Figure 2:**
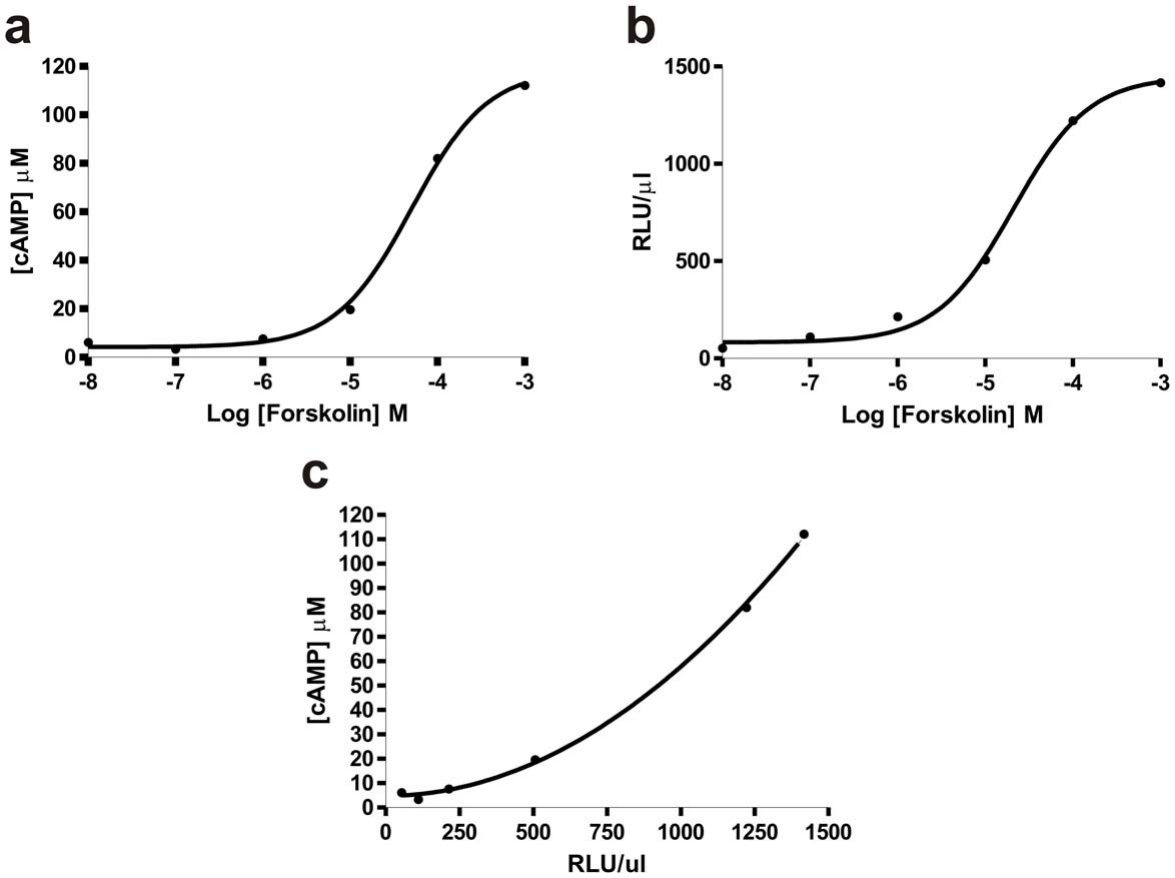
Validating the cAMP biosensor. A HEK293 cell line expressing the Glosensor™ cAMP biosensor under a tetracycline inducible promoter (FLP-IN™ system; Invitrogen) was generated. The reporter was validated by determining the effect of known doses of forskolin on levels of cAMP, determined by ELISA (**a**) and Glosensor bioluminescence (**b**). Data show mean for 2 (ELISA) and 3 (luminescence) separate experiments each of which contained samples in triplicate. Fits show sigmoidal dose response curves of the form y = a + b/1+10^(c-x)^ where a = bottom, b = top-bottom and c =  LogEC50, and yield EC50 values of 51 and 21 µM for ELISA and luminescence assays respectively. (**c**) A comparison of cAMP concentration and RLU for each forskolin concentration was used to infer the relationship between these parameters. This could be fit by a first order polynomial (R^2^ value of 0.999), suggesting that, under these conditions, cAMP concentration can therefore be estimated from RLU as follows: [cAMP] µM  = 4.785 + (0.0003971a) + (0.000005261â2) where a  =  RLU/µl.

A HEK293 cell line expressing the Glosensor™ cAMP biosensor under a tetracycline inducible promoter (FLP-IN™ system, Invitrogen; Glosensor, Promega; [20]) was generated. This biosensor is a modified luciferase that carries the cAMP binding B domain from the RIIβ subunit of cAMP-dependent protein kinase A (PKA). The Glosensor™ region was excised from the pGlosensor plasmid (Promega) and cloned into pcDNA5/FRT/TO, then co-transfected with pOG44 into FLP-IN™-293 cells. Isogenic stable cell lines were selected and maintained with 100 µg/ml hygromycin and 10 µg/ml blasticidin. Cells were maintained at 37°C in Dulbecco's modified Eagle's medium (DMEM), 4,500 mg/ml D-glucose, sodium pyruvate and L-glutamine (Sigma) with 10% foetal bovine serum (Sigma) in a 5% CO_2_ atmosphere. Following incubation with 300 ng/ml tetracycline, these cells showed repeatable and robust increases in raw luminescence (RLU) when stimulated with a range of forskolin concentrations (Figure 2).

The reporter was validated by determining the effect of known doses of forskolin on levels of cAMP, determined by ELISA and Glosensor bioluminescence, without any addition of phosphodiesterase inhibitors. Dose response curves of forskolin could be fit by both methods by sigmoidal dose response curves of the form y = a + b/1+10^(c-x)^ where a = bottom, b = top-bottom and c =  LogEC50, and yield EC50 values of 51 and 21 µM for ELISA and luminescence assays respectively, confirming the sensitivity of the bioassay. The relationship between cAMP and Glosensor response could then be inferred and fit by a first order polynomial (R^2^ value of 0.999), suggesting that, under these conditions, cAMP concentration can therefore be estimated from RLU as follows:

[cAMP] µM  = 4.785 + (0.0003971a) + (0.000005261â2)

where a  =  RLU/µl.

### Light response assays

7.5 x 10^3^ FLP-IN™-293 Glosensor cells in each well of solid white 96 well plates were transfected with plasmids containing opsin based receptors using Lipofectamine 2000 (Invitrogen) in serum-free medium for 6 hours. Immunocytochemistry revealed a transfection efficiency of ∼50% using this method. A stable cell line was constructed using a linearised plasmid of pcDNA3-HpaI JellyOp and additionally maintained with 400 µg/ml G418. Following transfection (or plating for the stable line), cells were then incubated for 16 hours with 300 ng/ml tetracycline and 10 µM retinaldehyde (9-*cis* retinal or all*-trans* retinal; Sigma-Aldrich) in CO_2_ independent medium without phenol red (L-15, Invitrogen), with 10% FCS. Beetle luciferin potassium salt (Promega) reconstituted in 10mM HEPES buffer was added to a final concentration of 2mM luciferin. All procedures following transfection of cells with opsins were carried out in dim red light.

Raw luminescence units (RLU) in cells were recorded at 37°C with 1s resolution with a top-read 3mm lens in a Fluostar Optima plate reader (BMG Labtech), with cycles of either 30s or 60s and gain of 3600. Following 30 mins equilibration, cells were subjected to repeated flashes from an electronic camera flash (The Jessop Group Ltd, UK; or forskolin application for cAMP biosensor validation) followed by recovery periods where RLU was recorded. Luminescence recordings were analysed with Optima (BMG Labtech) software and Microsoft Office Xcel. All experiments comprised cells plated and treated in triplicate. The average triplicate value for the five minutes prior to light treatment was used to normalise individual well values of the triplicate that were then averaged, except where baseline levels were examined. In the latter case, individual values were normalised to the triplicate average baseline RLU readings for mock transfected cells. Prism (Graphpad) software was used for all statistical analyses.

### Light response assays of stably expressing FLP-IN™-293 Glosensor JellyOp cells

7.5×10^4^ FLP-IN™-293 Glosensor JellyOp cells in each well of solid white 96 well plates were incubated with tetracycline and retinaldehyde as for temporal responses resulting from transient transfections above. In addition, for one experiment, 4 x 10^4^ FLP-IN™-293 Glosensor JellyOp cells were plated per well following pre-coating the wells with 0.01% poly-L-lysine (Sigma-Aldrich) in PBS for 2 hours. Cells were incubated as before and then treated with vehicle or 100 µM MDL2330A (Tocris Bioscience) for 15 mins before luminescence recordings as above.

An irradiance response curve was created for stable JellyOp expressing cells as follows. Cells were exposed to 10s of light from an array of white LEDs (Low energy floodlight, Palmer Riley Electricals UK) covered with layers of neutral density filter (Lee Filters, UK). Cells were left to recover and luminescence measured for 15 minutes and then exposed to a further light pulse brighter by 1 log unit, and this was repeated up to an irradiance of 4.66 mW cm^−2^. Spectral irradiance was measured with a spectroradiometer (Bentham Scientific).

### Immunocytochemistry

3.5 x 10^5^ cells/ml FLP-IN Glosensor cells were seeded onto coverslips and transfected with receptor constructs as above. 3.5 x 10^5^ cells/ml FLP-IN Glosensor JellyOp cells were seeded onto coverslips in red light. Following transfections, cells for MAPK labelling were incubated for 16 hours with 9-*cis* retinal in L-15 medium with 0.5% FCS and no phenol red. Cells were incubated with 100 ng/ml pertussis toxin (PTX, Tocris) for 16 hours, 10 µM U73122 (Tocris) for 30 mins, 100 µM MDL2330A for 15 mins or L-15 only. Dark treated cells were then washed with ice cold PBS and fixed with ice-cold 4% paraformaldehyde (PFA) for 30 mins. Simultaneously, light-treated plates were subject to 2 or 15 minutes exposure to a white LED array (The Litebook Company Ltd, Canada, 2.6 mW cm^−2^ at the level of the cells) and fixed as above. For labelling of the 1D4 epitope, cells were incubated for 16 hours with 9-*cis* retinal in L-15 medium with 10% FCS and fixed in the dark.

Fixed cells were blocked and permeabilised with 0.1% Triton-X 100, 5% BSA, 2% normal goat serum in PBS. Phospho-p44/42 MAPK antibody (CST; #9101) was used at a concentration of 1∶500 and rhodopsin 1D4 monoclonal antibody (Thermo Scientific Pearce) at 1∶1000. Phospho-MAPK labelled cells were incubated with a biotinylated secondary antibody and ABC streptavidin (Vectalabs) complex and developed with a DAB/Nickel chloride kit (Vectalabs). 1D4 labelling was visualised with Alexa555-conjugated secondary anti-mouse IgG antibody (Molecular Probes) at 1∶1000 and cells were mounted with Vectashield with DAPI (Vectalabs). Brightfield images of MAPK labelling were taken with an Axioskop upright microscope with Axiovision CCD camera and software. Fluorescent images were taken on a Nikon Eclipse upright 90i confocal microscope using 405nm and 543nm laser lines.

## Results

### OptoXR-driven responses have poor reproducibility

**Figure 3:**
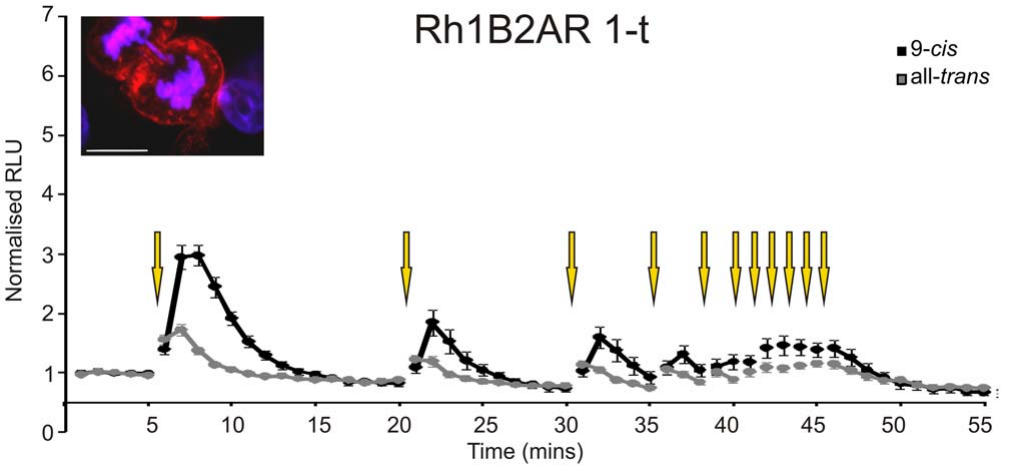
Real time analysis of a standard OptoXR response. Light induced changes in cAMP biosensor (Glosensor) luminescence in HEK293 cells transiently transfected with Rh1B2AR 1-t. Data for cells incubated with 9 *cis* retinaldehyde (black) or all *trans* retinaldehyde (grey) are shown; yellow arrows depict presentation of light flash. Data points show mean ± SEM n = 7. Inset shows immunocytochemical staining for the 1D4 epitope (in red, alexa 555 secondary antibody) of HEK293 cells expressing Rh1B2AR 1-t. DAPI shown in blue, scale bar  = 10 µm.

**Figure 4:**
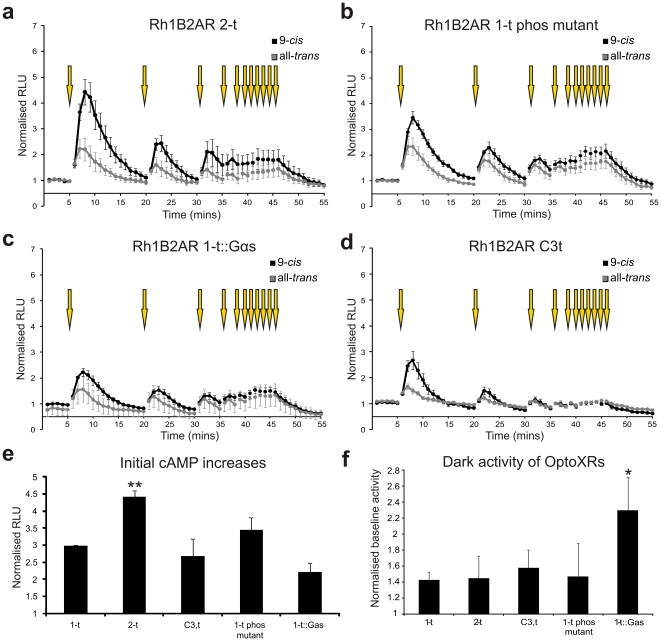
Real time analysis of OptoXR variant responses. (**a–d**) Light induced changes in cAMP biosensor (Glosensor) luminescence in HEK293 cells transiently transfected with Rh1B2AR 2-t (**a**) Rh1B2AR 1-t phos mutant (**b**), Rh1B2AR 1-t::Gαs (**c**) or Rh1B2AR C3,t (**d**). Luminescence was measured for 1 second every minute and _ormalized to baseline dark levels. Yellow arrows depict presentation of light flash. (**e**) Quantification of peak response amplitude for the first light flash following incubation with 9 *cis* retinaldehyde reveals that only the Rh1B2AR 2-t chimera shows an improved response amplitude compared to the Rh1B2AR 1-t OptoXR (one-way ANOVA with Dunnett's post hoc comparison to Rh1B2AR 1-t p<0.01). (**f**) None of these chimera exhibited reduced dark activity (luminescence prior to light exposure, _ormalized to mock transfected control cells), and indeed this parameter was significantly increased in cells expressing Rh1B2AR 1-t::Gαs when preincubated with 9 *cis* retinaldehyde (one-way ANOVA with Dunnett's post hoc comparisons to mock transfections as control group, p<0.05). *p<0.05, **p<0.01. Data show mean ± SEM (n≥4) shown for cells preincubated overnight with either 9-*cis* retinaldehyde (black) or all *trans* retinaldehyde (grey).

**Figure 5:**
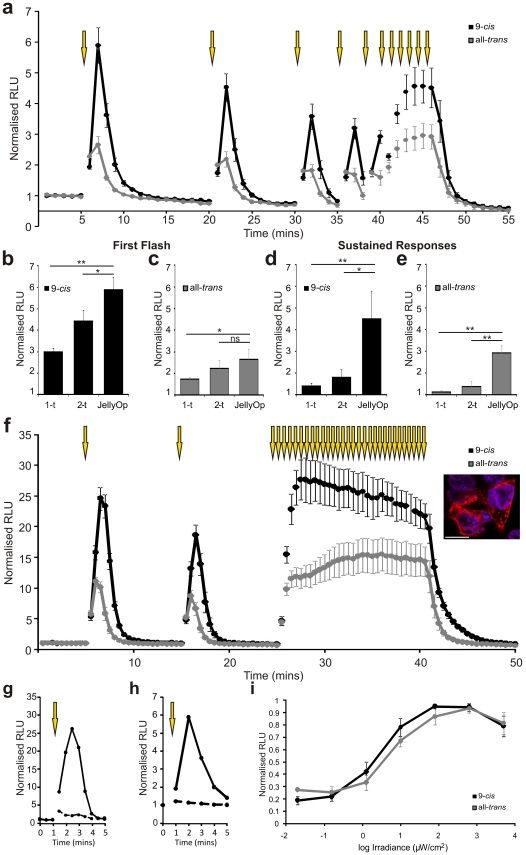
JellyOp driven light responses. (**a**) Light induced changes in cAMP biosensor luminescence (depicted _ormalized to baseline before light exposure) in HEK293 cells transiently transfected with JellyOp (n = 5). (**b,c**) The peak increase in luminescence following the first light flash was significantly greater for 9-*cis* pretreated cells expressing JellyOp than either the Rh1B2AR 1-t (1-t) or Rh1B2AR 2-t (2-t) chimera. It was also significantly greater for all-*trans* pretreated cells expressing JellyOp than Rh1B2AR 1-t. (**d,e**) Sustained responses were further enhanced, with luminescence over 5 minutes of light exposure (flashes once per minute) reaching a plateau significantly higher in JellyOp than either Rh1B2AR chimera. (**f**) Tracking changes in luminescence with higher temporal resolution (reading every 30s) in HEK293 cells stably transfected with JellyOp revealed high magnitude responses that could be sustained over 15 minutes of repeated stimulation (n = 4). Inset shows immunocytochemical staining for 1D4 epitope (red) in JellyOp expressing HEK293 cells. Nuclei stained blue with DAPI; scale bar 10 µm. (**g**) Cells stably expressing JellyOp show markedly repressed responses to a light flash when treated with 100 µM MDL2330A (adenylate cyclase inhibitor; dashed line), n = 1 (**h**) cAMP biosensor luminescence responses in HEK293 cells transiently transfected with JellyOp (continuous line) are abolished in the JellyOp lysine mutant (dashed line), n = 1 (**i**) Irradiance response curves for cAMP reporter activity in cells stably expressing JellyOp and induced with 10s white light (LED) pulses. RLU values are _ormalized to the peak response (n = 3). **A**-**I** show cells pre-incubated with either 9-*cis* (black) or all-*trans* (grey) retinaldehyde; mean ± SEM; yellow arrows depict timing of light flash. Data in **b**-**e** were analysed using one-way ANOVA with Dunnett's post hoc comparisons to JellyOp as control group, *p<0.05, **p<0.01; n≥4.

Measuring light-dependent cAMP accumulation in optogenetic studies presents a technological challenge. Standard end-point assays fail to capture one of the essential features of optogenetics, the ability for repeated and temporally defined activation. However, real-time assays based upon fluorescent calcium reporters or FRET reporters, which are now in standard use to measure cAMP [21], [22], [23], [24], are inappropriate because the excitation light for the fluorescence reporter will likely also activate the optogenetic photopigment. Here, we hoped to avoid these limitations by using a luciferase-based cAMP reporter, to provide a live-cell readout of this second messenger without impacting receptor activity (Figure 2, Figure 3, Figure 4, Figure 5). We started by transiently transfecting HEK 293 cells carrying this reporter with a humanised version of a published OptoXR, comprising the trans-membrane and extracellular elements of rod opsin fused to the intracellular components of the β2-adrenergic receptor (termed here Rh1B2AR 1-t; Figure 1). When these cells had been pre-incubated with 9-*cis* retinal, a single brief flash of light induced a rapid and reversible increase in luminescence (Figure 3). The magnitude of this response was robust. Thus, at its peak there was a 3.07±0.2 (mean ± SEM) fold increase in luminescence over background. We estimate a transfection efficiency of ∼50% from immunocytochemistry (see Methods; Light response assays). Using the formula derived from the relationship between Glosensor RLU responses to forskolin and measured cAMP values stimulated by forskolin, the peak cAMP light response by the Rh1B2AR 1-t receptor if transfection efficiency is corrected to 100% was ∼5.15 µM. This compares to around 7.27 µM cAMP produced in response to 1 µM forskolin or 18.48 µM cAMP produced in response to 10 µM forskolin in these cells. Rh1B2AR 1-t expressing cells pre-incubated with all-*trans* retinaldehyde did also show a light response (presumably because the HEK 293 cell line, unlike primary kidney cells, expresses RPE65 allowing it to generate *cis*-isoforms of retinaldehyde [25], [26]), but the associated increase in luminescence was much smaller than in the 9-*cis* retinaldyehyde condition (Figure 3).

The potentiating effect of exogenous 9-*cis* retinaldehyde on the OptoXR light response is consistent with the known dependence of rod opsin on *cis*-isoforms of retinaldehyde. On this basis, however, the effect is predicted not to survive repeated light exposure. This indeed was the case. We found that the light response of cells expressing the published Rh1B2AR 1-t rapidly diminished under these conditions. Thus, the peak response amplitude to a second brief flash was reduced by about 50%, with subsequent responses further diminished. Indeed, by the 5^th^ flash, responses were very small, indicating that the potentiating effect of the exogenous *cis*-retinaldehyde had been largely lost (Figure 3).

We generated a panel of structural variants of the Rh1B2AR chimera in an attempt to improve the magnitude of the OptoXR response. We aimed to obtain a reasonable response amplitude even in the absence of *cis*-retinaldehdyes and/or under repeated light exposure (Figure 4). One, in which the first cytoplasmic loop of rod opsin was retained (Rh1B2AR 2-t; Figure 4a), significantly improved the initial light response (peak RLU) following pre-treatment with 9-*cis* retinaldehyde (one-way ANOVA with Dunnett's post hoc comparison to Rh1B2AR 1-t, p<0.01, figure 4e). However, none of them overcame the fundamental limitation in response reproducibility.

### Improved responses in cells expressing JellyOp

The family of animal opsins contains members who are less susceptible to bleach than rod opsin, and one of these from the box jellyfish *Carybdea rastonii* has recently been shown to couple to Gαs in vitro [17]. We therefore next determined whether this opsin (termed JellyOp here) would allow us to overcome the poor reproducibility and sustainability of responses driven by Rh1 based OptoXRs.

When reconstituted overnight with 9-*cis* retinaldehyde, cells transiently transfected with JellyOp showed a 5.9±0.5 fold increase in cAMP reporter activity following a single light flash (Figure 5a&b). This is significantly greater than the best response we achieved with Rh1-based OptoXRs (from Rh1B2AR 2-t; p<0.05 one tailed t-test). Interestingly, the kinetics of the JellyOp response were also increased, with a sharper decline in luminescence suggesting that this receptor may allow finer temporal control of the second messenger. The real advantage of using JellyOp was, however, in the response to repeated stimulation. Thus, JellyOp-driven responses to subsequent flashes were only modestly reduced, and a series of 5 flashes maintained cAMP at a much higher level than that obtainable with OptoXRs (one-way ANOVA with Dunnett's post-hoc test, p<0.01; Figure 5d). The implication that JellyOp is able to support high amplitude cAMP responses without exogenous *cis*-retinaldehyde was confirmed by the activity of cells pre-incubated with all-*trans* retinal (Figure 5c). In these cells, response amplitude showed no sign of decreasing under repeated stimulation and, if anything, increased. Similarly, the JellyOp response under these conditions was much greater than that of OptoXRs (one-way ANOVA with Dunnett's post-hoc test, p<0.001, Figure 5e).

Basal levels of luminescence were modestly increased in cells expressing JellyOp. Thus, cells preincubated with 9-*cis* retinaldehyde had 1.21±0.1 times greater luminescence in the dark than untransfected controls, and 1.81±0.16 times greater luminescence with all-*trans* retinaldehyde. This likely represents genuine basal (dark) activity of the receptor, although given the high photosensitivity of JellyOp (see below), light produced by the luciferase reporter may also have driven some receptor photoactivation.

### JellyOp sensory characteristics

In order to further characterise the sensory characteristics of JellyOp in HEK293 cells, we generated a cell line stably expressing this opsin. The first notable feature of this cell line was the magnitude of light responses. We found that a single brief light pulse could increase luminescence by 24.47±2.22 (n = 5) fold in cells pre-incubated with 9-*cis* retinaldehyde (approx. 7.63 µM cAMP). Moreover, we were able to maintain this level of reporter activity for at least 15 mins with repeated stimulation (Figure 5f).

Consistent with the view that JellyOp-dependent increases in luminescence reflect opsin-based activation of adenylate cyclase activity via a Gαs cascade, we found that the light response of these cells was repressed by an adenylyl cyclase inhibitor (MDL2300A) and absent when a JellyOp mutant lacking the lysine critical for chromophore binding was used (Figure 5g,h). Treatment with inhibitors of canonical Gi or Gq cascades (pertussis toxin, a Gi inhibitor or U73122, a PLC inhibitor), however, did not have an effect on the light response (data not shown).

The most commonly used optogenetic tools require very bright light for activation [10], [27], [28]. We next determined whether this was also true for JellyOp by describing the irradiance dependence of the cAMP response in the stable cell line. We found a measurable increase in luminescence could be induced by 10s pulses of white light (LED source) at irradiances ≥1 µW/cm^2^ (Figure 5i). For reference, light levels at bench-top in our laboratory are typically around 200 µW/cm^2^. In fact this likely overestimates the light required to activate JellyOp because much of the energy produced by the white LED will be inefficiently absorbed by the pigment. JellyOp is maximally sensitive to light of around 500nm in wavelength [17], and we estimate that responses could be elicited by as little as 11 log photons/cm^2^/s (<100 nW/cm^2^) if a 500nm narrow band light source were employed. The increase in reporter luminescence at these threshold irradiances corresponds to that induced by around 1 µM forskolin.

### Light dependent mitogen activated protein kinase (MAPK) signalling

**Figure 6:**
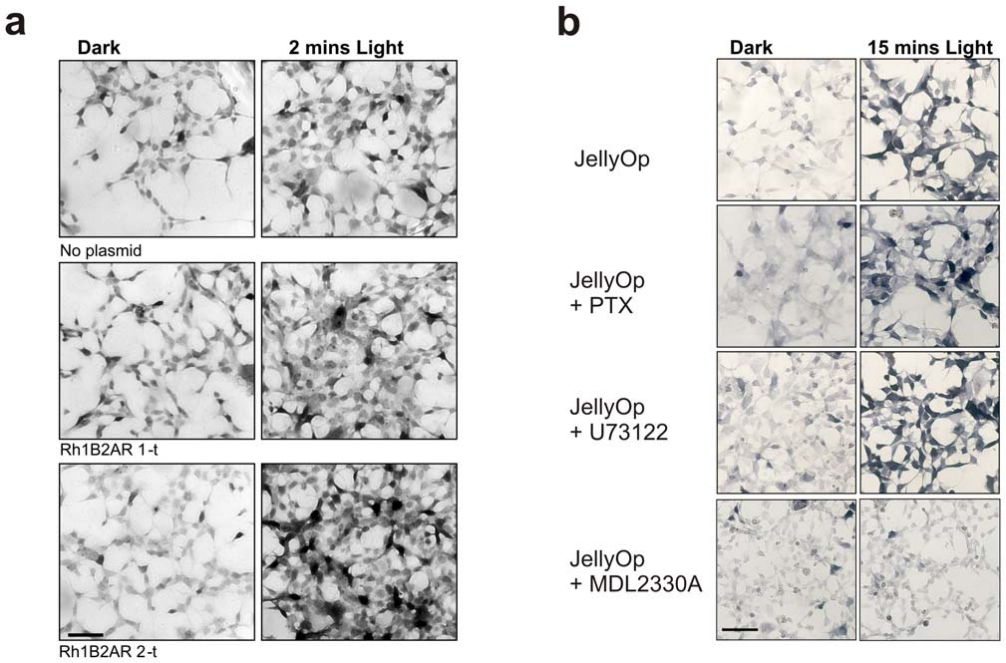
MAPK phosphorylation. Immunohistochemical labelling of phosphorylated MAPK (grey-black stain produced with horseradish peroxidise and diaminobenzidine) in HEK293 cells preincubated with 9-*cis* retinaldehyde and exposed to either 2 minutes of white light or kept in the dark. (**a**) OptoXRs elicit a light dependent increase in MAPK phosphorylation. Photomicrographs for untransfected cells and cells expressing each of the OptoXRs were taken with the same exposure settings. Scale bar  = 50 µm. (**b**) Phosphorylated MAPK in HEK293 cells expressing JellyOp exposed to the dark or 15 mins of light. Cells labelled ‘+PTX’ were treated with 100ng/ml pertussis toxin (a Gi inhibitor); ‘+U73122’ with 10 µM U73122 (a Gq inhibitor); ‘+MDL2330A’ with 100 µM (adenylate cyclase inhibitor). MDL inhibits light-induced MAPK phosphorylation following both 2 mins (not shown) and 15 mins of light. The increase in phosphorylation therefore appears to be due to a Gs dependent pathway.

GPCR signalling is not restricted to conventional Gα/second messenger pathways but may also involve Gβγ and G-protein independent (β-arrestin) pathways that can lead to MAPK phosphorylation and activation of other cascades, typically following a longer time course than the conventional pathways [29]. We found that both JellyOp and all of the OptoXRs used in this study drove a light dependent increase in MAPK phosphorylation in HEK293 cells (Figure 6). Thus, there was a marked increase in immunoreactivity for phospho-MAPK, following 2 or 15 minutes exposure to a white LED based light source (Litebook™; 2.6 mW cm^−2^) in HEK293 FLP-IN Glosensor cells transiently expressing any of these receptors. In agreement with data from cAMP Glosensor recordings, treatment of cells expressing JellyOp with inhibitors of canonical Gi or Gq cascades (pertussis toxin or U73122) did not result in measurable reduction of MAPK phosphorylation following light treatment. Conversely, treatment with an adenylate cyclase inhibitor inhibited MAPK phosphorylation at both shorter and longer (15 mins) time points. This indicates that MAPK phosphorylation under these conditions is downstream of an increase in cAMP rather than attributed to G protein independent pathways following light activation.

## Discussion

The conventional approach to manipulating GPCR activity is pharmaceutical. However, drugs have a number of well-recognised limitations. Off-target effects on tissues and cell types other than those of interest occur with even the most localised delivery systems, while the kinetics of drug delivery and clearance impose a limit on the temporal resolution with which systems can be manipulated. Optogenetics provides an exciting opportunity to overcome these problems by using a ‘ligand’ (light), which can be applied with µm/µsec spatiotemporal resolution, to activate a receptor targeted to specific cell types using the tools of genetic engineering. The ultimate expression of this technology will be to impose natural patterns of GPCR activity. Realising this ambition will, however, require optogenetic tools that allow both repeatable and sustained activation. There is abundant evidence that this level of control is physiologically relevant. Thus, cAMP signalling in units as small as sub-cellular compartments has been described [30], [31], [32], while pulsatile and oscillatory patterns of cAMP concentration are common and can elicit different downstream responses than continuous cAMP elevations [22], [33], [34], [35]. The data presented here suggests that this is achievable using JellyOp, a bleach resistant visual opsin from the box jellyfish *Carybdea rastonii*.

Optogenetic control of Gαs signalling has previously been achieved using chimeric receptors comprising light absorbing elements of mammalian rod opsin and G-protein interaction domains from other GPCRs [9], [10]. Such OptoXRs have been used successfully to regulate cAMP and Ca^2+^ in vitro and in vivo, but the reproducibility and sustainability of activation have not been rigorously interrogated. Here we first set out to achieve this using a new luciferase-based cAMP biosensor to track live cell responses driven by OptoXRs. Our data reveal intrinsic limitations to the currently available rhodopsin/β2 adrenergic receptor chimera (Rh1B2AR 1-t). Thus, although light drove a clear induction of cAMP in HEK293 cells expressing this receptor, responses were low amplitude without preincubation with *cis*-retinaldehyde. We were able to achieve modest improvements in this aspect of performance by changing chimera design. However, the more important deficit was in response reproducibility, and none of our revised OptoXRs were able to recover the precipitous decline in response amplitude over repeated stimulation. This aspect of OptoXR behaviour is predicted by the bleaching characteristics of rod opsin, which requires a ready supply of *cis*-retinaldehyde to remain photosensitive in its native environment. As the enzymatic machinery required to generate large amounts of *cis*-retinaldehyde is restricted to the eye in mammals, this could be an important limitation to optogenetic application. Some researchers have applied exogenous *cis*-retinaldehyde when using rod opsin-based tools for optogenetic control [7]. Our data suggest that this represents only a partial solution to the problem, as its effectiveness should rapidly decline under repeated or continuous light exposure.

Our data suggest that JellyOp overcomes these critical limitations to allow reproducible and sustained cAMP responses. These features are consistent with published evidence that JellyOp is intrinsically bleach-resistant. Many animal opsins have two photo-interconvertible stable states binding either *cis*- or *trans*-isoforms of retinaldehyde. This enables them to remain photosensitive under repeated/continuous light exposure. The photobiology of JellyOp is incompletely understood, but Koyanagi et al (2008 [17]) were able to show that when purified JellyOp was reconstituted with 11-*cis* retinaldehyde, light exposure led to creation of a thermostable pigment binding all-*trans* retinaldehyde and absorbing visible wavelengths. In order to be fully bleach resistant, further light exposure should reconstitute the 11-*cis* isoform. Koyanagi et al (2008 [17]) did not report this final step in bleach recovery for JellyOp, but this may be due to inadequacies in the *in vitro* environment. Our data support that view. The relatively poor performance of our rod-opsin based OptoXRs except in the presence of exogenous *cis*-retinaldehdye highlights how important such bleach-resistance is likely to be for *in vivo* application. A new generation of optogenetic tools based upon bleach-resistant animal opsins could then be contemplated.

JellyOp is the only animal opsin known to be Gαs coupled. This has enabled us to use it to achieve control of Gαs signalling without OptoXR-like modifications of putative G-protein interaction domains. Nevertheless, future work could usefully concentrate on structural modifications to the receptor. The intracellular surface of GPCRs is responsible not only for defining G-protein specificity, but also for targeting the receptor to specific cellular compartments; defining the rate of receptor deactivation; and coupling to non-canonical signalling pathways. It should therefore be possible in future to adjust these aspects of JellyOp activity by changing its intracellular domains. This potential to approximate multiple aspects of native GPCR activity sets JellyOp aside from other approaches directly targeting cAMP production. A group of microbial photoactivated adenylate cyclases (PACs) have been used to achieve this latter goal [28], [36], [37], [38], although it is not yet clear whether these PACs approach the repeatability/sustainability of JellyOp signalling.

One most notable feature of JellyOp is its high sensitivity. We achieved pharmacological levels of cAMP induction with a white light stimulus as low as 1 µW/cm^2^. This is many orders of magnitude below that required to activate the microbial light gated ion channels used so widely in neuroscience [27] and at least 10x below the threshold for the most sensitive currently available optogenetic tools [38], [39], [40]. As this means that JellyOp can be activated using simple, inexpensive, white light sources, it makes it a very accessible technology.
